# Effects of Using a Text Message Intervention on Psychological Constructs and the Association Between Changes to Psychological Constructs and Medication Adherence in People With Type 2 Diabetes: Results From a Randomized Controlled Feasibility Study

**DOI:** 10.2196/30058

**Published:** 2022-04-29

**Authors:** Yvonne Kiera Bartlett, Andrew Farmer, Nikki Newhouse, Lisa Miles, Cassandra Kenning, David P French

**Affiliations:** 1 Manchester Centre for Health Psychology School of Health Sciences University of Manchester Manchester United Kingdom; 2 Nuffield Department of Primary Care Health Sciences University of Oxford Oxford United Kingdom; 3 Division of Population Health, Health Services Research & Primary Care University of Manchester Manchester United Kingdom

**Keywords:** medication adherence, type 2 diabetes mellitus, behavior change techniques, text messaging, feasibility studies, diabetes, medication, digital health

## Abstract

**Background:**

Poor adherence to oral medications is common in people with type 2 diabetes and can lead to an increased chance of health complications. Text messages may provide an effective delivery method for an intervention; however, thus far, the majority of these interventions do not specify either a theoretical basis or propose specific mechanisms of action. This makes it hard to determine how and whether an intervention is having an effect. The text messages included in the current intervention have been developed to deliver specific behavior change techniques. These techniques are the “active ingredients” of the intervention and were selected to target psychological constructs identified as predictors of medication adherence.

**Objective:**

There are 2 aims of this study: (1) to assess whether a text message intervention with specified behavior change techniques can change the constructs that predict medication adherence behaviors in people with type 2 diabetes and (2) to assess whether changes to psychological constructs are associated with changes in self-reported medication adherence.

**Methods:**

We conducted a randomized controlled, 6-month feasibility trial. Adults prescribed oral medication for type 2 diabetes (N=209) were recruited from general practice and randomized to either receive a text message–based intervention or care as usual. Data were analyzed with repeated measures analysis of covariance and Spearman rho correlation coefficients.

**Results:**

For 8 of the 14 constructs that were measured, a significant time-by-condition interaction was found: necessity beliefs, intention, maintenance self-efficacy, recovery self-efficacy, action control, prompts and cues, social support, and satisfaction with experienced consequences all increased in the intervention group compared to the control group. Changes in action self-efficacy, intention, automaticity, maintenance self-efficacy, and satisfaction with experienced consequences were positively associated with changes in self-reported medication adherence.

**Conclusions:**

A relatively low-cost, scalable, text message–only intervention targeting medication adherence using behavior change techniques can influence psychological constructs that predict adherence. Not only do these constructs predict self-reported medication adherence, but changes in these constructs are correlated with changes in self-reported medication adherence. These findings support the promise of text message–based interventions for medication adherence in this population and suggest likely mechanisms of action.

**Trial Registration:**

ISRCTN Registry ISRCTN13404264; https://www.isrctn.com/ISRCTN13404264

## Introduction

Poor adherence to oral treatments is common in people with type 2 diabetes [1,2]. When such medication is taken suboptimally, blood glucose control can be poorer, leading to greater risk of developing complications [3], which can affect the heart, eyes, blood vessels, nerves, and other organs [4]. This has implications for people with diabetes and those supporting them, and is also associated with increased costs for health services [5].

An updated Cochrane review of 182 interventions to improve medication adherence concluded that “current methods of improving medication adherence for chronic health problems are mostly complex and not very effective,” [6] and therefore, new approaches are needed to address this problem. Technology-based interventions may have the potential to improve medication adherence at scale at low unit cost. Thus far, no single approach has been identified as being the most effective or ineffective [7]. However, providing brief messages such as text messages is particularly promising, as text messages have the advantage of already being widely adopted and low-cost [8]. In a recent systematic review and meta-analysis of interventions based on multiple behavior targets, such as diet, exercise, and medication adherence, text messages were found to be effective in reducing levels of blood glucose for people with type 2 diabetes [9]. However, only 1 intervention included in this review exclusively targeted medication adherence, and this consisted only of medication reminder texts. This approach is unlikely to be sufficient unless the only barrier to adherence is forgetting, which excludes intentional nonadherence and the person with diabetes taking an active role in making adjustments to their medication regimen around their daily life, both of which have been observed in this population [10]. In a review specifically looking at medication adherence, it was concluded that although brief messages show promise, more high-quality evidence is needed [11]. Specifically, very few of the included studies stated an explicit theoretical basis, and of those that did, none discussed the results in relation to this theory.

Having an explicit theoretical basis provides clarity in terms of the proposed mechanism of action of an intervention; that is, what the intervention is intended to do and how. This helps with both the development and evaluation of digital health interventions. In development, having a logic model that describes how an intervention is intended to work can help designers choose what elements to include. By defining the constructs the intervention is targeting, components such as behavior change techniques (BCTs) that are hypothesized to affect these constructs can be chosen.

BCTs have been described as the “active ingredients” of an intervention and include techniques such as problem solving. There is currently a taxonomy of 93 BCTs with descriptions that can be used by intervention designers [12]. Using standardized BCTs as intervention components allows for easier comparison across interventions, greater transparency in what the intervention consists of, and the potential to use systematic reviewing and statistical analyses to identify effective BCTs across trials. For example, a meta-regression of randomized controlled trials that used either short messages and/or interactive voice recognition software to support medication adherence for cardio-metabolic conditions identified that the BCT, information about health consequences, was positively associated with effect size [13].

In terms of evaluation, an explicit logic model helps with understanding why a change in behavior either has or has not happened [14]. As an example, intention to take medication has been identified as a psychological construct that is important in changing medication adherence behavior [15]; therefore, an intervention designer might include BCTs thought to have an effect on intention, such as information about health consequences. Once the intervention has been delivered, evaluating the mechanism of action (ie, whether intention was changed as hypothesized) would help to explain the presence or absence of change in medication adherence behavior. This in turn would affect the further development of the intervention. Different adjustments would be made to the intervention if the proposed change in intention did not occur or if intention changed but this did not result in changes to medication adherence. Although links have been proposed between BCTs and psychological constructs, they are not often tested empirically [16]. Testing each link in the chain from BCT, to construct, to change in behavior would provide the strongest explanation of how an intervention functions and add to the understanding of medication adherence behavior. Furthermore, defining and evaluating components within an intervention and how they are proposed to work is considered key to facilitating the accumulation of evidence related to digital health interventions [17].

**Figure 1:**
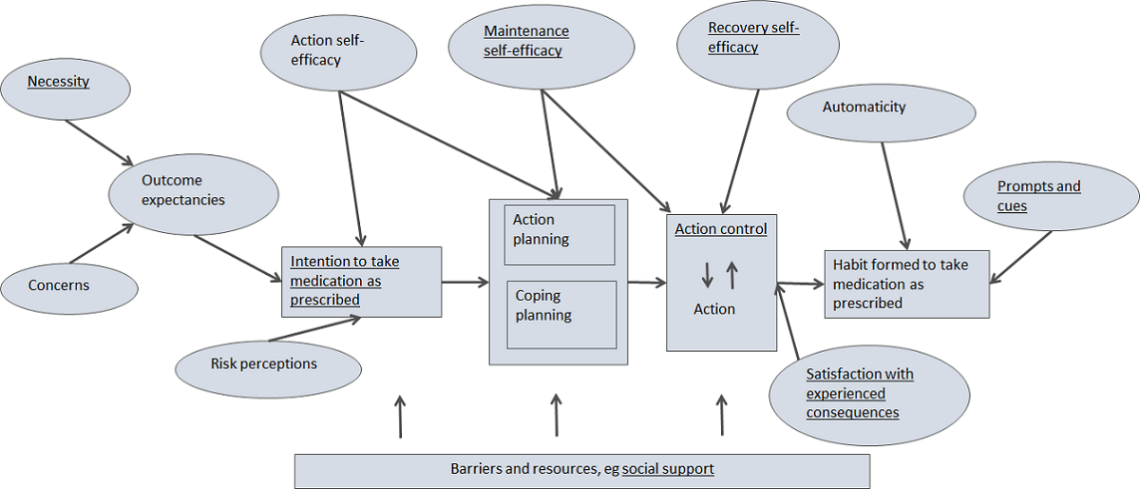
Proposed theoretical model based on the Health Action Process Approach [17]. Underlined constructs indicate those that were significantly increased in the intervention group vs. the control group.

Prior to this study, a rapid systematic review was conducted that identified psychological constructs related to medication adherence from systematic reviews and meta-analyses as well as BCTs that target these constructs [15]. Although many of these psychological constructs have been found to predict medication adherence, there is not yet evidence that changing these constructs produces change in medication adherence. This evidence of causation is urgently needed. According to the evidence in this rapid systematic review, a conceptual model of the intervention was developed based on the Health Action Process Approach [18], with additions to reflect the evidence synthesized. This model conceptualizes medication adherence as a process, from forming an intention, acting on that intention, and then monitoring and adjusting these actions until adherence becomes habitual (see Figure 1). The approach taken follows the recommendations of the updated Medical Research Council (MRC) framework for development and evaluation of complex interventions to develop such logic models to facilitate process analyses [19,20].

A library of text messages was developed to deliver specified BCTs that target multiple constructs relevant to this model (see Table 1). The included constructs relate to both intentional and nonintentional nonadherence.

Our previous research has confirmed that the messages in the library are good examples of the BCTs they were written to represent and are acceptable to the target population [21]. We are therefore confident that the messages can deliver the intended BCTs; however, at present we do not know if these BCTs will have the proposed effects on psychological constructs or if changes to these psychological constructs will be related to changes in medication adherence. Hence, there was a need for this formative study to indicate if changes are required to the intervention before causal links are further explored in an efficacy trial powered to conduct this analysis. To thereby test the mechanism of action of the intervention, relevant psychological constructs were measured as part of a randomized controlled feasibility trial [22] in order to answer the following 2 research questions: (1) Does a BCT-based brief message intervention produce changes in psychological constructs relative to control group?; (2) Are changes in psychological constructs correlated with changes in medication adherence?

**Table 1:** Example messages with associated BCTs (replicated from Bartlett et al [21]).

Target and category of message	BCT^a^/belief or concern	Example messages
Medication adherence, BCT	1.4^b^ Action planning	“Plan when, where and how you are going to take your medication.”
Medication adherence, BCT	15.1^b^ Verbal persuasion about capability	“If you are struggling with your diabetes tablets then don't worry, you will be able to master it in time. You will get on top of it.”
Medication adherence, BCT	7.1^b^ Prompts/cues	“It can be difficult to remember to take your tablets. Why not set an alarm to remind you to take them?”
Medication adherence, beliefs, and concerns	Health care system–related concerns	“Lots of questions? Check who the best person to see might be.”
Diet management	Signposting	“Stuck for new ideas? You can search recipes for mains, desserts and snacks online at Diabetes.org.uk.”

^a^BCT: behavior change technique.

^b^Numerical identifiers from the taxonomy [12].

## Methods

### Recruitment

**Figure 2:**
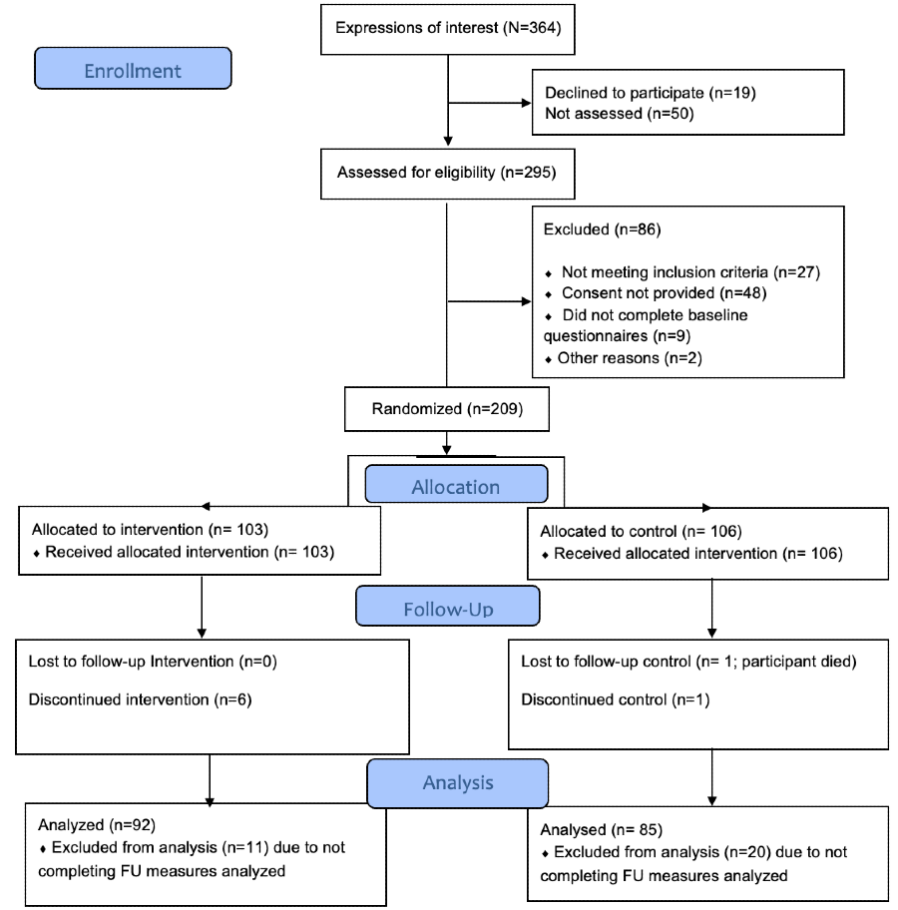
Support Through Mobile Messaging and Digital Health Technology for Diabetes (SuMMiT-D) feasibility CONSORT (Consolidated Standards of Reporting Trials) flow diagram.

Participants were recruited from 16 general practices in England between January 2019 and June 2019. Potentially eligible patients were contacted about the study by the practice and invited to send a text message to express interest. On receipt of the text message, further information about the study was given either online or by post, and eligibility was assessed by the researchers by phone. Eligible patients were those who were ≥35 years of age, able to use a mobile phone to send and receive text messages, and taking oral medication for type 2 diabetes (including lipid and blood pressure–lowering medications for diabetes). Patients taking oral medication either with or without concomitant insulin were eligible. Patients who had been admitted to hospital in the previous 3 months with hypo- or hyperglycemia, were pregnant, were within 3 months postpartum, were planning a pregnancy within the trial, or had a serious medical condition that, in the opinion of the investigator, made them unable to take part were ineligible. Informed consent was given either online or by post. See Figure 2 for the CONSORT (Consolidated Standards of Reporting Trials) flowchart.

### Design

Data were collected for a 6-month, parallel-group, randomized controlled feasibility trial as part of the Support Through Mobile Messaging and Digital Health Technology for Diabetes (SuMMiT-D) program of work. Participants were randomized in a 1:1 ratio to the intervention or control arm. Randomization was completed through a validated secure web-based program (Sortition) using a nondeterministic minimization algorithm to ensure groups were balanced for age, study site, gender, duration of diabetes, and number of medications. The allocated intervention was then delivered directly through an online platform. Aside from those conducting qualitative interviews and the engineering team, all other research team members and health care staff were blinded. For further details of the design and development, see the protocol paper [22].

### Ethical Approval

Ethical approval for the study was granted by National Health Service (NHS) West of Scotland Research Ethics Committee 05 (reference number 18/WS/0173). The trial is registered in the ISRCTN registry (ISRCTN13404264).

### Intervention

Participants in the intervention group were sent up to 4 text messages per week for 6 months. There were 2 categories of messages: (1) those targeting medication adherence based on BCTs identified as relevant for this population [15] that have previously been confirmed as representing the intended BCT and being acceptable to the target population [21] and (2) those targeting diet and physical activity, introduced as a response to feedback during the development process, indicating that a broader view of diabetes self-management may benefit engagement. Messages in category 2 provided information from and links to credible sources such as the Diabetes UK or NHS Choices website. All messages were sent at a preferred time (AM or PM), and participants were able to text back “like” or “dislike” after any message received. For messages targeting medication adherence, texting “like” doubled the chance a future message would come from the same BCT as the message that had been liked, while texting “dislike” halved the chance of a future message coming from the same BCT as the disliked message. Texting “like” or “dislike” following messages targeting diet or physical activity did not result in any change.

### Control

Participants in the control group received 1 message per month for 6 months thanking them for their participation in the study; this was in addition to their usual care.

### Assessments

Assessments were completed online or on paper. At baseline, participants completed a demographic questionnaire and provided their postcode, and at baseline and 6 months, participants completed the 5-item Medication Adherence Report Scale (MARS) [23,24] and a health psychology questionnaire. The 5 items on the MARS are nonadherent behaviors, and thus participants respond by indicating how true each statement is for them on a 5-point scale from “always true” to “never true.” One item referred to nonintentional nonadherence, “I forget to take my diabetes medicines,” while the other 4 items measured intentional nonadherence.

The hypothesized mechanisms of action questionnaire was developed for this study and measured key constructs targeted by the messages (see Table 2). Two items were used to measure each of the following fourteen constructs: action self-efficacy, necessity, concerns, intention, automaticity, maintenance self-efficacy, recovery self-efficacy, action planning, coping planning, action control, prompts and cues, social support, satisfaction with the experienced consequences of behavior, and risk perception. The 28 items were sourced or adapted from previously developed questionnaires where possible and were phrased to specifically relate to taking diabetes tablets as prescribed (see Table 2). All questions were answered using a 5-point Likert scale with the anchors strongly disagree, disagree, neither agree nor disagree, agree, and strongly agree. For further details on additional measures taken but not reported here, see Farmer et al [22].

**Table 2:** Properties of the psychological construct scales.

Construct	Example item	Interitem correlation at baseline				Paper adapted from
		Correlation coefficient (R_s)_	*n*	*P* value		
Action self-efficacy	“I am confident that I can take my diabetes tablets as prescribed”	0.82	203	<.001		Schwarzer et al [25]
Necessity	“My health in the future will depend on my diabetes tablets”	0.53	203		<.001	Horne et al [26]
Concerns	“I sometimes worry about the long-term effects of my diabetes tablets”	0.19	203		.007	Horne et al [26]
Intention	“I will take my diabetes tablets as prescribed every day over the next 3 months”	0.81	204		<.001	Presseau et al [27]
Automaticity	“Taking my diabetes tablets as prescribed is something I do without thinking”	0.50	199		<.001	Gardner et al [28]
Maintenance self-efficacy	“I am confident that I am able to take my diabetes tablets as prescribed even when something disrupts my routine”	0.54	200		<.001	Greer et al [29]
Recovery self-efficacy	“If I don’t take my diabetes tablets for any reason, I am confident that I am able to start taking them again even if I feel no different to when I was not taking them”	0.63	201		<.001	Greer et al [29]
Action planning	“I have made a detailed plan about exactly where to take my diabetes tablets”	0.72	200		<.001	Greer et al [29]
Coping planning	“I have made a detailed plan for how to deal with unpleasant side effects of taking my diabetes tablets as prescribed”	0.46	200		<.001	Greer et al [29]
Action control	“During the last 4 weeks I consistently monitored when, where, and how I took my diabetes tablets”	0.23	201		.001	Sniehotta et al [30]
Prompts and cues	“I use things around me to help me to take my diabetes tablets as prescribed (e.g. notes, phone reminders)”	0.63	202		<.001	N/A^a^
Social support	“I have felt supported in taking my diabetes tablets as prescribed”	0.29	202		<.001	Presseau et al [27]
Satisfaction with experienced consequences	“I am content with what I have experienced as a result of taking my diabetes tablets”	0.75	203		<.001	Baldwin et al [31]
Risk perception	“I feel very at risk of developing complications, or experiencing worsening of existing complications from my diabetes if I do not take my tablets”	0.64	201		<.001	N/A

^a^N/A: not applicable.

### Analysis

The index of multiple deprivation (IMD) is a measure of relative deprivation used by the English government. Areas (32,844 across England) are ranked according to a variety of domains, including income, employment, health, and crime, and then the ranked list is divided into deciles [32]. Participants’ postcodes were used to identify their IMD decile (1 missing, postcode invalid). Descriptive statistics were used to describe age, gender, and IMD, while a *t* test or chi-squared test was used to assess differences in these variables between those who did and did not complete follow-up assessments. Responses were coded in the following fashion according to the MARS: never true=5, rarely true=4, sometimes true=3, often true =2, and always true=1. Thus, higher scores would be associated with better self-reported adherence. The hypothesized mechanisms of action questionnaire was scored as follows: strongly agree=5, agree=4, neither agree nor disagree=3, disagree=2, and strongly disagree=1. Thus, higher scores would be associated with higher levels of the construct (eg, greater action control, higher self-efficacy, or higher concerns). Construct scores were calculated by summing the scores for both items. Interitem correlations were calculated (see Table 2).

#### Research Question 1: Does a BCT-Based Brief Message Intervention Produce Changes in Psychological Constructs Relative to a Control Group?

Repeated-measures analysis of covariance (ANCOVA) was conducted for each construct, with time as a within-subject factor at 2 levels (baseline and 6-month follow-up); group (intervention or control) as a between-subject factor; and age, gender, and IMD included as covariates. As a sensitivity analysis, univariate ANCOVA for each construct were conducted, with construct at follow-up as the dependent variable; gender and experimental group as fixed factors; and construct at baseline, age, and IMD as covariates.

#### Research Question 2: Are Changes in Psychological Constructs Correlated With Changes in Medication Adherence?

Standardized residual change scores were calculated using linear regression for each construct (baseline to follow-up) and MARS (baseline to follow-up). Spearman rho correlation coefficients were then calculated to assess the relationship between change in standardized residuals for each construct and change in self-reported adherence.

## Results

### Participants

Participants (N=209) had a mean age of 63.4 years (SD 10.16), were 41.1% (86/209) female, and were recruited from all 10 of the IMD deciles with a mean of 6.38 (SD 2.73). Thirty-one participants were excluded from this analysis, as they did not complete the follow-up assessments analyzed here, and one participant died prior to follow-up. There were no significant differences between groups in age, gender, or IMD at baseline or between those who completed and did not complete the questionnaire measures at follow-up (see Table 3).

**Table 3:** Demographics.

Variable	Overall, mean (SD) (N=209)	Intervention, mean (SD) (N=103)	Control, mean (SD), (N=106)	Between-group differences at baseline^a^	Differences between completers (n=177) and noncompleters (n=31)—hypothesized mechanisms of action questionnaire assessing constructs^a^	Differences between completers (n=168) and noncompleters (n=40) in MARS^ab^
Age, (years)	63.44 (10.16)	63.47 (10.64)	63.42 (9.72)	.98	.96	.91
Female	86 (41.1)^c^	42 (40.8)^c^	44 (41.5)^c^	.51	.36	.22
IMD^d^ deciles^e^	6.38 (2.73)	6.10 (2.71)	6.65 (2.73)	.15	.55	.98

^a^Values in this column are *P* values.

^b^MARS: 5-item Medication Adherence Report Scale.

^c^Values in this cell are number and percentage.

^d^IMD: index of multiple deprivation.

^e^n=208: 1 postcode was incorrect and could not by mapped onto the IMD.

#### Research Question 1: Does a BCT-Based Brief Message Intervention Produce Changes in Psychological Constructs Relative to a Control Group?

A significant interaction between time and experimental group was seen in necessity (*P*=.009), intention (*P*<.001), maintenance self-efficacy (*P*=.03), recovery self-efficacy (*P*=.02), action control (*P*=.001), prompts and cues (*P*=.002), social support (*P*<.001), and satisfaction with experienced consequences (*P*=.002; see Table 4). All effects were such that the constructs increased between baseline and follow-up in the intervention group compared to the control group. Of the covariates (age, gender, IMD), only age had a significant effect on any constructs. Age had a significant effect on the model for necessity, action planning, social support, and satisfaction with experienced consequences. Sensitivity analysis treating baseline variables as covariates rather than as within-subject factors showed aligned significant or nonsignificant effects for 12 of the 14 constructs measured. Previously significant effects on recovery self-efficacy (*P*=.12) and maintenance self-efficacy (*P*=.30) were not replicated.

**Table 4:** Repeated-measures analysis of covariance effect of the text message intervention on psychological constructs.

Item	Control, mean (SD)		Intervention, mean (SD)		Main effect time, *F* test (*df*), *P* value^a^	Interaction time×group, *F* test (*df*), *P* value^a^	Significant covariates, Covariate: *F* (*df*), *P* value^a^
	BL^b^	FU^c^	BL	FU			
Action self-efficacy	8.87 (1.37)	8.65 (2.05)	8.55 (1.82)	8.80 (1.49)	.88	.10	N/A^d^
Necessity	7.67 (1.71)	7.73 (1.83)	7.44 (1.63)	8.15 (1.53)	.72	*F*_1,165_=7.03, .009	Age: *F*_1,165_=7.12, .008
Concerns	5.73 (1.67)	5.73 (1.84)	5.85 (1.65)	5.56 (1.64)	*F*_1,163_=4.17, .043	.18	Age: *F*_1,163_=7.58, .007
Intention	9.10 (1.06)	8.77 (1.62)	8.61 (1.51)	9.14 (1.24)	.11	*F*_1,164_=14.31, <.001	N/A
Automaticity	7.51 (1.71)	7.51 (1.95)	7.12 (1.85)	7.59 (1.77)	.65	.06	N/A
Maintenance self-efficacy	8.48 (1.34)	8.29 (1.44)	7.91 (1.59)	8.19 (1.44)	.58	*F*_1,159_=4.68, .032	N/A
Recovery self-efficacy^e^	8.55 (1.27)	8.56 (1.56)	8.10 (1.56)	8.67 (1.33)	.65	*F*_1,161_=5.50, .02	N/A
Action planning	6.94 (2.21)	7.24 (2.10)	6.88 (2.01)	7.49 (1.96)	.72	.32	Age: *F*_1,161_=4.51, .04
Coping planning	5.88 (1.83)	6.32 (1.66)	6.05 (1.63)	6.70 (1.77)	.11	.42	N/A
Action control	7.10 (1.79)	7.05 (1.81)	6.99 (1.74)	7.88 (1.59)	.12	*F*_1,160_=10.80, .001	N/A
Prompts and cues	5.38 (2.07)	5.59 (2.09)	4.91 (1.75)	6.26 (1.99)	.22	*F*_1,160_=10.31, .002	N/A
Social support	4.71 (1.55)	4.74 (1.71)	4.95 (1.75)	6.11 (1.65)	.24	*F*_1, 160_=16.40, <.001	Age: *F*_1,160_=4.80, .03
Satisfaction with experienced consequences	7.78 (1.91)	7.60 (1.68)	7.47 (1.81)	8.16 (1.62)	.45	*F*_1, 162_=9.77, .002	Age: *F*_1,160_=4.73, .03
Risk perception	8.08 (1.53)	8.78 (1.76)	8.07 (1.44)	8.22 (1.61)	.70	.53	N/A

^a^Test statistic and degrees of freedom are only reported for *P* values <.05 in this column.

^b^BL: baseline.

^c^FU: follow-up.

^d^N/A: not applicable (no significant covariates were found).

^e^Potentially there is less confidence in this result as recovery self-efficacy was significantly different between groups at baseline such that intervention (mean 8.07, SD 1.54) was higher than the control (mean 7.98, SD 1.52; t_199_=2.59; *P*=.01).

#### Research Question 2: Are Changes in Psychological Constructs Correlated With Changes in Medication Adherence?

Standardized residual change scores in 5 of the 14 psychological constructs were significantly positively correlated with those in self-reported medication adherence, such that increases in the construct represented improvements in medication adherence action self-efficacy (*r*_s_=0.28; n=149; *P*=.001), intention (*r*_s_=0.20; n=149; *P*=.02), automaticity (*r*_s_=.33; n=143; *P*<.001), maintenance self-efficacy (*r*_s_=0.30; n=147; *P*<.001), and satisfaction with experienced consequences (*r*_s_=0.16; n=148; *P*=.05).

## Discussion

### Principal Results

In this analysis we have shown that first, provision of a text message–based intervention using behavior change techniques results in improvements to multiple psychological constructs compared to usual care. Second, we have identified that changes in psychological constructs are correlated with changes in self-reported medication adherence. These findings support the hypothesized mechanisms of action that are amenable to change through a low-cost, scalable intervention, and that when changed, may have an effect on medication adherence in people with type 2 diabetes. These findings, although tentative, provide a strong base on which to progress to a full efficacy trial.

### Strengths and Weaknesses

The intervention messages use a wide variety of BCTs that are thought to target different points in the process of adherence. The incorporation of a wide variety of techniques, including some BCTs that have not been applied in this context previously, constitutes one of the strengths of this intervention, as this represents a new way to approach medication adherence. However, a corresponding weakness is that this could make looking at each individual link between BCTs and constructs more difficult, as several BCTs might have affected the same construct.

These findings have shown that this intervention can have an effect on multiple constructs that may influence people at different points in the process of improving medication adherence from forming an intention, acting on that intention, to monitoring and adjusting these actions until adherence becomes habitual (see Figure 1). In addition, the intervention targets sources of both intentional and nonintentional nonadherence. The potential to affect change in people wherever they are in the process of improving medication adherence is a definite strength, from which a wide range of people with type 2 diabetes can benefit. In this feasibility study, we were not powered to conduct a formal mediation analysis, however this is planned for the definitive trial which is now underway (ISRCTN 15952379).

Medication adherence has been self-reported here using the MARS. In the future, we plan to take additional measures of adherence (eg, from medical records) so that the relationships between these constructs, self-reported adherence, and adherence measured through more direct means can be explored. Future work could also explore the use of objective measures of medication adherence, such as urine samples [33]. Overall, this study provides a more detailed picture of the potential mechanism of action for this intervention, which can be used to support development of further interventions for this target behavior.

The eventual aim is that the brief text message intervention can be delivered at scale, through general practice. In terms of future scalability, basing the intervention solely on text messages is highly cost-effective. Recent research has indicated combining text messages with interactive voice recognition can be an effective intervention for medication adherence in this population [34]; there is further evidence that incorporating tailoring can make interventions more effective [13]. Any additional components and technology required above and beyond text messages, and any additional complexity may limit the eventual scalability of the intervention. Understanding the unique effects of nontailored text messages alone in the first instance is useful, as this is the lowest-cost approach. Additional elements could then be added to the intervention where they would provide the most benefit and when the evidence is clearer on which conditions tailoring can be optimally applied to.

The measures of psychological constructs used in this study were by necessity brief to minimize participant burden. There is increasing recognition that high questionnaire burden in trials has undesirable consequences, such as reducing recruitment, increasing dropout in low socioeconomic status or minority ethnic groups, and producing unintended reactions to this measurement [35]. It was therefore necessary to use a questionnaire developed for this study. Items from pre-existing scales were used when possible, and the correlations for the majority of items were considered moderately or strongly correlated [36]. However, there were 3 constructs with weak correlations between items (concerns, action control, and social support), and we aim to improve these items for our future research. An alternative approach could be to use this preliminary work to identify specific constructs of interest and measure a smaller number of constructs with validated scales.

### Future Development

Future research could use these findings for the following purposes: to investigate those constructs that did not change in this instance, and whether there are more effective BCTs to target these constructs than those used here; to explore those constructs where changes did not correlate with changes in medication adherence; and to improve the measurement of constructs where correlations between the 2 items were weak. This work would help to gather additional information that could be used to optimize interventions for this population.

The findings reported indicate that certain constructs are both amenable to change by text message and, when changed, are associated with changes in self-reported medication adherence (eg, intention, maintenance self-efficacy, and satisfaction with experienced consequences). These constructs could indicate the importance of continued feedback and adjustment within medication adherence interventions; following initial changes to intention, it may be necessary to support people to maintain and highlight the positive effects of changes made to support satisfaction with continued adherence. BCTs that target these constructs may be useful for focusing on future research into medication adherence.

This feasibility trial was not powered to look at direct effects of the intervention on the outcome. The findings do provide a clear indication of the potential value of an intervention such as this, but in the planned trial of this intervention participant numbers will be sufficiently high to ascertain efficacy of the intervention and allow for mediation analysis to further explore the potential mechanisms of action suggested here. By identifying likely mechanisms of action of the intervention beforehand, efficacy results will be more easily interpreted. In addition, with a larger sample, it may be possible to conduct subanalysis to explore whether changes in constructs are associated with particular participant characteristics, and this could provide evidence to inform future tailoring strategies. Incorporating tailoring increases the complexity of an intervention and potentially reduces the scalability. However, if future tailored interventions were compared with this nontailored intervention, an evidence base could be built on how to tailor in the most effective way, which would only introduce additional complexity where there is likely to be maximum benefit.

### Conclusions

A text message intervention based on behavior change techniques can affect psychological constructs that are correlated with medication adherence. The use of a logic model enabled clear proposed mechanisms of action to be defined and tested. Future research can explore these potential mechanisms further to improve the understanding of adherence behavior and intervention design.

## Appendix

Multimedia Appendix 1 CONSORT-eHEALTH checklist (V 1.6.1).

## Abbreviations

ANCOVA: analysis of covariance
BCT: behavior change technique
CONSORT: Consolidated Standards of Reporting Trials
IMD: index of multiple deprivation
MARS: Medication Adherence Report Scale
MRC: Medical Research Council
NHIR: National Institute for Health Research
NHS: National Health Service
SuMMiT-D: Support Through Mobile Messaging and Digital Health Technology for Diabetes
